# *Lactiplantibacillus plantarum* Y40 Ameliorates *Salmonella* Infection via PPARγ-Mediated Regulation of Fatty Acid Metabolism in Mice

**DOI:** 10.3390/microorganisms14061210

**Published:** 2026-05-27

**Authors:** Lifang Gu, Hui Zhang, Jinyan Yu, Jiaxuan Jiang, Meicun Hou, Kun Wang, Shenglong Liang, Jingru Lu, Jing Ju, Haoyu Liu, Xinan Jiao, Yunzeng Zhang

**Affiliations:** 1 Jiangsu Co-Innovation Center for Prevention and Control of Important Animal Infectious Diseases and Zoonoses, Yangzhou University, Yangzhou 225009, China; gulifang1997@163.com (L.G.); zhanghui@163.com (H.Z.); eve_421@163.com (J.Y.); jjx2019096109@163.com (J.J.); 15820873480@163.com (M.H.); wkun878573123@163.com (K.W.); 17851971057@163.com (S.L.); 18068611426@163.com (J.L.); 17851375001@163.com (J.J.); 2 Jiangsu Key Laboratory of Zoonosis, Yangzhou University, Yangzhou 225009, China; 3 Joint International Research Laboratory of Agriculture and Agri-Product Safety of the Ministry of Education, Yangzhou University, Yangzhou 225009, China; haoyu.liu@yzu.edu.cn; 4 Key Laboratory of Prevention and Control of Biological Hazard Factors (Animal Origin) for Agrifood Safety and Quality, Ministry of Agriculture of China, Yangzhou University, Yangzhou 225009, China; 5 College of Animal Science and Technology, Yangzhou University, Yangzhou 225009, China; 6 College of Bioscience and Biotechnology, Yangzhou University, No. 48 Wenhui East Road, Yangzhou 225009, China

**Keywords:** *Lactiplantibacillus plantarum*, *Salmonella* Typhimurium, PPARγ, fatty acid metabolism

## Abstract

*Salmonella* Typhimurium disrupts intestinal homeostasis by inducing inflammation and metabolic dysregulation during infection. Although probiotic-mediated protection against *Salmonella* infection has been widely reported, the underlying host-targeted mechanisms remain incompletely understood. Here, we investigated the protective effects of wild badger-derived *Lactiplantibacillus plantarum* Y40 against *S.* Typhimurium SL1344 infection in mice, focusing on host metabolic regulation. We found that Y40 pretreatment significantly alleviated infection-induced colonic inflammation and epithelial barrier disruption. Transcriptomic analysis revealed that SL1344 infection induced an immune-dominant transcriptional profile, characterized by activation of inflammatory pathways and suppression of metabolic processes, particularly fatty acid metabolism. In contrast, Y40 pretreatment reprogrammed host gene expression by attenuating inflammatory responses while restoring metabolic pathways. Targeted serum fatty acid profiling demonstrated that Y40 reversed infection-induced reductions in serum free fatty acids and normalized fatty acid composition, including restoration of oleic acid (C18:1N9C) and linoleic acid (C18:2N6). Mechanistically, Y40 upregulated peroxisome proliferator-activated receptor gamma (PPARγ) and its downstream targets (*FASN*, *ACC1*, and *SCD1*), which were suppressed by SL1344 infection. Pharmacological inhibition of PPARγ in MC38 cells abolished the protective effects of Y40. Collectively, these findings establish that *L. plantarum* Y40 protects against *Salmonella* infection by activating PPARγ-dependent fatty acid metabolism, thereby maintaining intestinal barrier integrity and limiting inflammation.

## 1. Introduction

*Salmonella enterica* subsp. *enterica*, particularly non-typhoidal serovars such as *S*. Typhimurium, represents one of the most significant foodborne pathogens, posing a severe threat to both the livestock industry and public health globally [1]. In livestock, *Salmonella* infection leads to enteric disease, growth impairment, and systemic dissemination, while contaminated animal-derived products represent a major source of human infection [1,2]. Despite extensive control efforts, *Salmonella* remains a persistent challenge. Indeed, non-typhoidal *Salmonella* causes tens of millions of human cases annually, with estimated costs exceeding $2–4 billion annually in the United States alone [3].

Upon entry into the host via contaminated food, water, or the fecal–oral route, *Salmonella* triggers robust host immune activation characterized by inflammatory cytokine production and epithelial barrier integrity disruption [4,5]. Beyond immune responses, accumulating evidence indicates that *Salmonella* actively reprograms host cellular metabolism to its advantage [6]. Recent studies highlight that *S*. Typhimurium, for example, subverts host metabolic pathways, notably by altering glycolysis and nutrient fluxes, to create a favorable intracellular niche [7,8]. Among these metabolic processes, lipid metabolism, particularly fatty acid (FA) pathways, has emerged as a critical regulator of infection outcomes [9]. Fatty acids are central to cellular energy and membrane homeostasis and serve as signaling molecules that link metabolism to immunity [10]. Commensal-derived fatty acids (such as short-chain and branched-chain fatty acids) can enhance epithelial barrier integrity and attenuate inflammation by activating receptors and intracellular pathways that inhibit NF-κB and other pro-inflammatory signals [11,12]. Conversely, dysregulation of FA metabolism tends to exacerbate inflammation and barrier dysfunction [13,14]. Collectively, these alterations foster an environment conducive to pathogen colonization and persistence.

The metabolic–immune processes are tightly coordinated by lipid-sensing nuclear receptors. Peroxisome proliferator-activated receptor gamma (PPARγ) is highly expressed in intestinal epithelial cells and immune cells, where it couples lipid metabolism to immune regulation [15]. Activated PPARγ promotes FA uptake and lipid storage while directly enhancing epithelial barrier function by upregulating the expression of tight-junction protein-encoding genes and anti-inflammatory-associated genes [16]. Conversely, impaired PPARγ signaling leads to dysregulated lipid metabolism, loss of barrier integrity, and heightened inflammatory cytokine production [17,18,19]. Indeed, PPARγ agonists (e.g., the antidiabetic drug pioglitazone) have been shown to restore tight junction expression and suppress TNF-α/IL-1β signaling in models of intestinal inflammation [20,21]. Notably, enteric pathogens may exploit this axis; *Salmonella* infection, for instance, is associated with the downregulation of PPARγ pathways, favoring a pro-inflammatory, catabolic host state [22]. In contrast, activation of PPARγ signaling normally restricts the expansion of pathogens like *Salmonella* by limiting luminal electron acceptors [23]. Thus, the modulation of PPARγ and FA metabolism represents a key battleground in enteric infection. However, the mechanisms by which probiotic intervention restores this balance during *Salmonella* infection remain incompletely understood.

Probiotics are known to confer protection against *Salmonella* and other enteric pathogens through diverse mechanisms, including competitive exclusion, the secretion of antimicrobial compounds, and the modulation of host immune responses [24]. An emerging theme is that probiotics can reprogram host metabolism to strengthen resistance [25]. Certain *Lactiplantibacillus plantarum* strains have been reported to beneficially modulate lipid metabolism and restore metabolic homeostasis under inflammatory conditions. For example, *L. plantarum* treatment in *Salmonella*-infected chickens restored disrupted tight-junction gene expression and significantly suppressed inflammatory mediators, including MPO, TNF-α homologs, IL-1β, and IL-6, compared to infected controls [26]. However, most studies to date have focused on microbial community shifts or canonical immune effects, leaving the integration of host metabolic pathways, particularly FA metabolism, within these protective mechanisms largely underexplored [27].

In our previous work, we isolated a strain of *L. plantarum* designated Y40 from a wild badger and demonstrated its potent protective efficacy against *Salmonella* infection in mice [28]. Based on the emerging concepts outlined above, we hypothesized that the benefits of Y40 involve the reprogramming of the host metabolic response to infection, specifically through lipid and PPARγ-mediated pathways. To test this hypothesis, we conducted a systematic analysis, performing transcriptomic profiling of colonic tissues from *Salmonella*-infected BALB/c mice with or without Y40 pretreatment, coupled with serum fatty acid profiling and in vitro cell assays. This multi-tiered approach aimed to elucidate how Y40 modulates the host’s metabolic–immune crosstalk during *Salmonella* challenge. Notably, the BALB/c mouse infection model used in this study was established without antibiotic pretreatment and therefore represents a systemic typhoid-like *Salmonella* infection model rather than acute colitis [29,30]. Nevertheless, this model has been widely used for evaluating probiotic-mediated protection against *Salmonella* infection and investigating host protective mechanisms relevant to livestock and poultry production systems [31,32,33].

## 2. Materials and Methods

### 2.1. Bacterial Strains and Cell Culture

*L. plantarum* Y40 was isolated from a fresh wild badger fecal sample and cultured in MRS broth (Guangdong Huankai Microbial Sci. & Tech. Co., Ltd., Guangzhou, China) at 37 °C for 10 h [28]. *S.* Typhimurium SL1344 was obtained from the Jiangsu Key Lab of Zoonosis at Yangzhou University (Yangzhou, China), and grown in LB broth (Sangon Biotech, Shanghai, China) at 37 °C with shaking (180 rpm) for 8 h.

Mouse colonic carcinoma MC38 cells were obtained from Hunan Fenghui Biotechnology (Changsha, China) and maintained in high-glucose Dulbecco’s Modified Eagle Medium (DMEM) (HyClone, Logan, UT, USA) supplemented with 10% fetal bovine serum (FBS) (Gibco, Grand Island, NY, USA) and 1% penicillin/streptomycin (Solarbio, Beijing, China) at 37 °C in a 5% CO_2_ humidified incubator.

### 2.2. Animal Experiments and Sample Collection

The animal study was conducted in accordance with the guidelines approved by the Jiangsu Administrative Committee of Laboratory Animals (SYXK-[Su]-20220044). Male specific pathogen-free (SPF) BALB/c mice (6–8 weeks old) were acquired from the Institute of Comparative Medicine at Yangzhou University. Only male mice were used to reduce sex-associated variability, as previous studies have demonstrated substantial sexual dimorphism in inflammatory, transcriptional, and metabolic responses to bacterial infection, including *Salmonella* challenge [34,35]. After a 5-day acclimation period, mice were randomly assigned into three groups (*n* = 8): control, SL1344, and Y40+SL1344. The Y40+SL1344 group was pre-treated with 10^8^ CFU Y40 for 7 days by gavage. On day 8, the SL1344 and Y40+SL1344 groups were challenged with 10^5^ CFU *S.* Typhimurium SL1344. The control group received an equivalent volume of sterile PBS (Beyotime Biotechnology, Shanghai, China) daily for 8 days. On day 6 post-infection, mice were euthanized under sterile conditions for collection of organs and colonic tissues; blood samples were collected by immediate postmortem cardiac puncture.

### 2.3. Quantification of SL1344 in Fecal and Tissue Samples

*Salmonella* burdens in fecal and colon samples were quantified using a qPCR assay targeting the invA gene, as previously described [28]. Briefly, the *invA* gene fragment [36] was cloned into the pMD19T plasmid vector (Takara Bio, Dalian, China) and transformed into *Escherichia coli* DH5α (Takara Bio, Dalian, China) for propagation. The extracted plasmid was serially diluted to generate a standard curve for absolute quantification. DNA extracted from fecal and colonic tissue samples collected at 6 dpi was amplified in parallel under identical qPCR conditions. Sample Cₜ values were interpolated against the standard curve to determine invA copy number per reaction and normalized to copies per gram of sample based on the initial sample mass and extraction volume. The liver, spleen, and kidney samples were harvested, weighed, and homogenized in sterile PBS. Serial dilutions of tissue homogenates were plated onto XLT4 agar (BD Difco, Franklin Lakes, NJ, USA) supplemented with streptomycin (100 μg/mL) (Shanghai Macklin Biochemical Co., Ltd., Shanghai, China). After incubation at 37 °C for 12 h, *Salmonella* colonies were counted and expressed as CFU per gram of tissue.

### 2.4. RNA Sequencing and Bioinformatics Analysis

Total RNA was extracted from colonic tissues using the SteadyPure Universal RNA Extraction Kit (Accurate Biotechnology, Changsha, China) according to the manufacturer’s protocol. RNA quality was assessed using NanoDrop 2000 (Thermo Fisher Scientific, Waltham, MA, USA) and Agilent Bioanalyzer 2100 (Agilent Technologies, Santa Clara, CA, USA). Libraries were constructed and sequenced on an Illumina NovaSeq 6000 platform (Illumina, San Diego, CA, USA) (6 Gb clean reads per sample) by Shanghai BIOZERON Co., Ltd (Shanghai, China). Raw reads were quality-filtered using fastp (quality threshold q > 20, length > 80 bp) [37], rRNA-derived reads were removed using SortMeRNA (version 4.3.4), and the remaining reads were aligned to the mouse reference genome (mm10) using HISAT2 [38]. Gene expression quantification was performed using HTSeq-count [39], and differential expression analysis was conducted with DESeq2 (FDR < 0.05, |log2 fold change| > 1) [40]. GO and KEGG pathway enrichment analyses were performed using Metascape (http://metascape.org/) [41], and GSEA [42] was conducted using GSEA 4.1.0 software. The RNA sequencing data have been deposited in the China National GenBank database (CNGBdb) under project ID CNP0009403.

### 2.5. Serum Fatty Acid Analysis

Whole blood was collected via cardiac puncture from mice. Serum was separated by centrifugation at 3500 rpm for 10 min at 4 °C and stored at −80 °C until analysis. Total free fatty acid (FFA) concentrations were determined using the Amplex Red Free Fatty Acid Assay Kit (Beyotime Biotechnology, Shanghai, China) following the manufacturer’s instructions. The fatty acid profiles were analyzed by gas chromatography-mass spectrometry (Thermo Trace 1300 GC-ISQ 7000 MS, Thermo Fisher Scientific, Waltham, MA, USA) using a TG-FAME capillary column (Thermo Fisher Scientific, Waltham, MA, USA). The oven temperature was initially set at 80 °C for 1 min, then increased at 20 °C/min to 160 °C and held for 1.5 min, then increased at 3 °C/min to 196 °C and held for 8.5 min, and finally increased at 20 °C/min to 250 °C and held for 3 min. The inlet and ion source temperatures were 250 °C and 300 °C, respectively. The split ratio was 8:1, and a 1 µL injection volume was used. Helium was used as the carrier gas at a constant flow of 0.63 mL/min. Mass spectrometry was performed using electron impact ionization at 70 eV with selected ion monitoring (SIM) mode. Fatty acid peaks were identified and quantified using Xcalibur software (version 3.0.63) based on standard curves generated from authentic standards.

### 2.6. In Vitro Infection and PPARγ Inhibition Assays

MC38 cells were seeded in 24-well plates at 4 × 10^5^ cells per well in high-glucose DMEM supplemented with 10% FBS (Gibco, Grand Island, NY, USA) (without antibiotics) and cultured to confluence. For the Y40+SL1344 group, 1 × 10^7^ CFU of *L. plantarum* Y40 cells were added to each well and incubated for 1.5 h. The cells were then washed with PBS and transferred to fresh medium. After that, *S.* Typhimurium SL1344 cells were added to *L. plantarum*-treated and untreated MC38 cells at a multiplicity of infection (MOI) of 20:1 and incubated for 1 h.

For PPARγ inhibition, cells were pretreated with 15 μM GW9662 (Beyotime Biotechnology, Shanghai, China) or DMSO (Sigma-Aldrich, St. Louis, MO, USA) vehicle control for 3 h prior to Y40 treatment in the Y40+SL1344 group.

### 2.7. RNA Extraction and Quantitative Real-Time PCR (qRT-PCR)

Total RNA was extracted from colon samples or cells using the SteadyPure Universal RNA Extraction Kit (Accurate Biotechnology, Changsha, China) and reverse-transcribed using the *Evo M-MLV* Reverse Transcription Kit (Accurate Biotechnology, Changsha, China). qRT-PCR was performed using gene-specific primers (Table S5) with β-actin as the internal reference. Relative gene expression was calculated using the 2^−ΔΔCt^ method [43].

### 2.8. Western Blot Analysis

The colon samples or cells were lysed in RIPA (Beyotime Biotechnology, Shanghai, China) buffer containing PMSF on ice for 20 min. Lysates were centrifuged at 12,000 rpm at 4 °C for 5 min, and supernatants were collected. The obtained protein concentration was measured by BCA protein concentration analysis kit (Beyotime Biotechnology, Shanghai, China). Proteins were separated by SDS-PAGE and then transferred to PVDF membranes (MilliporeSigma, Burlington, MA, USA). Membranes were then blocked with 5% non-fat powdered milk in Tris buffer containing 0.1% Tween-20 (TBST) for 2 h at room temperature. After blocking, membranes were incubated with primary antibodies against IL-1β (Cell Signaling Technology, Danvers, MA, USA), ZO-1, Occludin, and β-actin (HUABIO, Hangzhou, China), Claudin-1, TNF-α, and IL-6 (Proteintech, Wuhan, China), overnight at 4 °C followed by incubation with HRP-conjugated secondary antibodies (Beyotime Biotechnology, Shanghai, China). Finally, the membrane was reacted with an ultrasensitive kit (Beyotime Biotechnology, Shanghai, China), imaged on a Chemi Doc™ XRS + gel imaging system (Bio-Rad, Hercules, CA, USA), and densitometrically quantified using ImageJ (version 1.47).

### 2.9. Statistical Analysis

Statistical analysis was carried out using GraphPad Prism 9.0 (GraphPad, San Diego, CA, USA) unless otherwise noted. Quantitative data are represented by mean ± SD. Statistical significance was determined using Kruskal–Wallis test followed by Dunn’s post hoc test with Bonferroni correction for multiple comparisons, unless otherwise specified. Statistical significance was set at *p* < 0.05. All experiments included at least three biological replicates or independent experiments, as indicated in the figure legends.

## 3. Results

### 3.1. Protective Effects of Y40 Against SL1344 Infection Provide a Basis for Transcriptomic Profiling

**Figure 1:**
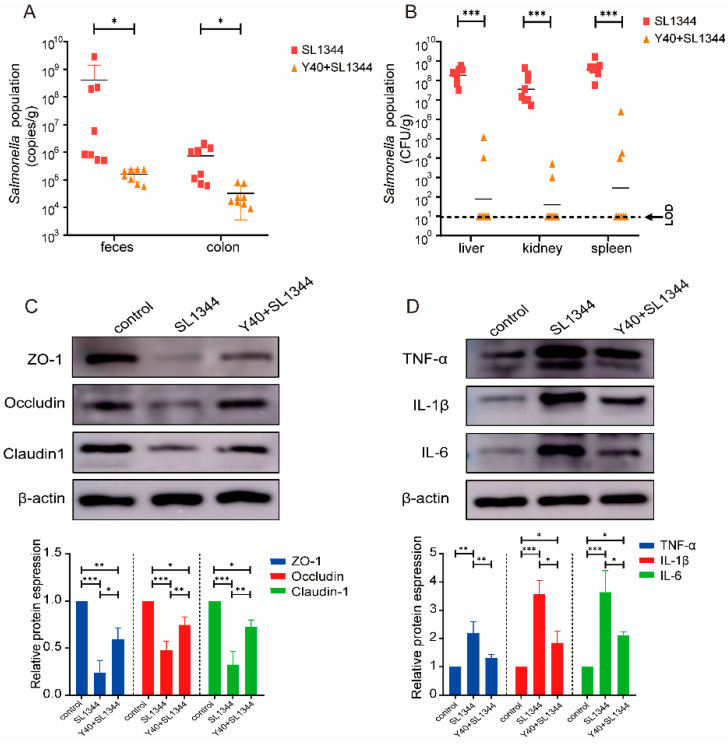
In vivo protective effects of *L. plantarum* Y40 against *S.* Typhimurium SL1344 infection. (**A**) *Salmonella* burdens in fecal and colonic tissues quantified by *invA*-based qPCR (*n* = 8 per group, Mann–Whitney U test). (**B**) Salmonella burdens in liver, spleen, and kidney determined by plate counting (CFU/g tissue, *n* = 8 per group, Mann–Whitney U test). (**C**) Representative Western blot (top) and densitometric quantification (bottom) of tight junction proteins (ZO-1, Occludin, and Claudin-1). (**D**) Representative Western blot (top) and densitometric quantification (bottom) of pro-inflammatory cytokines (TNF-α, IL-1β, and IL-6). Data in (**C**,**D**) are from *n* = 3 independent experiments (Kruskal–Wallis test with Dunn’s post hoc test). * *p* < 0.05; ** *p* < 0.01; *** *p* < 0.001. Error bars denote SD.

In the present study, mice infected with SL1344 (SL1344 group) exhibited marked body weight loss by day 6 post-infection compared with the control mice (control group), whereas Y40 pretreatment (Y40+SL1344 group) significantly attenuated this reduction, maintaining body weight comparable to that of uninfected controls (Figure S1). Moreover, SL1344 infection resulted in substantial *Salmonella* colonization in both intestinal and systemic tissues, including feces, colon, liver, spleen, and kidney. In contrast, Y40 pretreatment significantly reduced SL1344 burdens compared with the infected group (Figure 1A,B), indicating that Y40 effectively suppresses *Salmonella* colonization and systemic dissemination in vivo. Consistently, SL1344 infection induced pronounced colonic inflammation, as evidenced by increased expression of pro-inflammatory cytokines (TNF-α, IL-1β, and IL-6) and disruption of epithelial barrier integrity in the colon, indicated by downregulation of tight junction proteins (ZO-1, Occludin, and Claudin-1). In contrast, Y40 pretreatment effectively alleviated inflammatory responses and restored the expression of these barrier-associated proteins (Figure 1C,D). These results confirmed the protective effect of Y40 and provided a foundation for subsequent transcriptomic analysis.

RNA sequencing (RNA-seq) was then performed on colonic tissues from control, SL1344-infected, and Y40-pretreated infected mice to systematically characterize host transcriptional responses. A total of 415,997,244 paired-end reads (150 bp) were generated from nine samples, with an average of 46,221,916 reads (approximately 6.93 Gb) per sample. After removing rRNA-derived reads using SortMeRNA [44], an average of 79.13% of clean reads were retained, of which 94.41% were successfully mapped to the mouse reference genome (mm10). Differential expression analysis using DESeq2 identified differentially expressed genes (DEGs) between the SL1344 and control groups to assess host responses to infection, and between the Y40+SL1344 and SL1344 groups to evaluate the regulatory effects of Y40 pretreatment.

### 3.2. SL1344 Infection Reshapes Immune and Metabolic Transcriptional Profiles in the Colon

**Figure 2:**
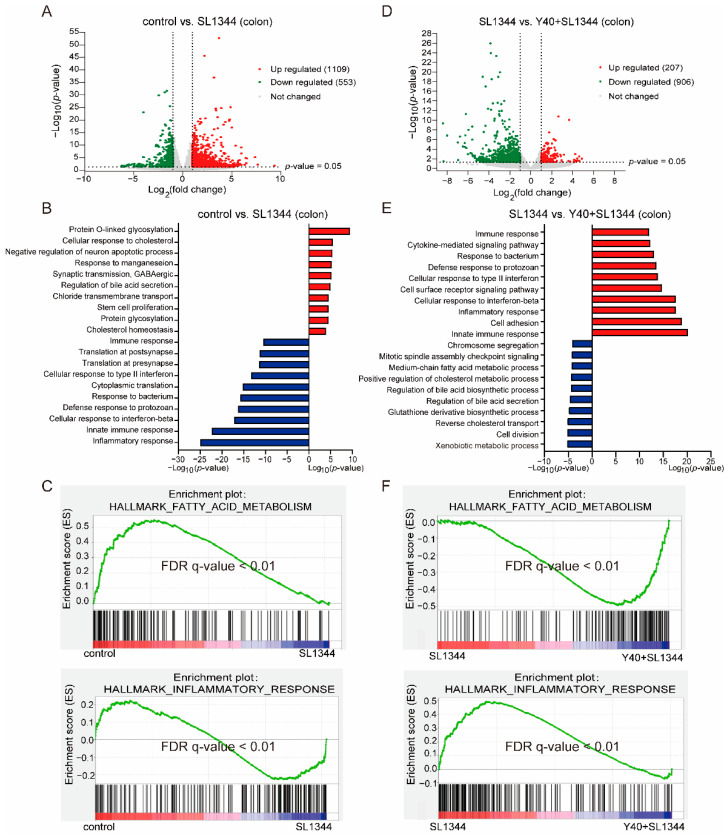
Systematic transcriptomic analysis of mouse colonic tissue following *S.* Typhimurium SL1344 infection and *L. plantarum* Y40 pretreatment. (**A**,**D**) Volcano plots depicting DEGs (control vs. SL1344; Y40+SL1344 vs. SL1344). (**B**,**E**) GO biological process enrichment of DEGs. (**C**,**F**) GSEA plots for fatty acid metabolism and inflammatory response. RNA-seq was performed using *n* = 3 colonic samples per group.

Venn analysis revealed that 18,085 genes were commonly expressed between the control and SL1344 groups, with 2019 and 648 genes uniquely expressed in the SL1344 and control groups, respectively. Differential expression analysis identified 1662 DEGs (FDR < 0.05 and |log2 fold change| > 1), including 1109 upregulated and 553 downregulated genes in the SL1344 group compared with controls (Figure 2A, Table S1), indicating a substantial remodeling of the colonic transcriptome following infection.

Gene Ontology (GO) enrichment analysis revealed that these DEGs were predominantly associated with immune response and epithelial homeostasis (Figure 2B). The most significantly enriched biological processes included inflammatory response, innate immune response, cellular response to interferon-beta, defense response to protozoan, and response to bacterium. Additionally, pathways related to epithelial barrier function, such as protein O-linked glycosylation and cholesterol homeostasis, were significantly altered, suggesting that SL1344 infection disrupts both immune balance and epithelial integrity.

KEGG pathway analysis further demonstrated that the upregulated genes were mainly enriched in immune-related pathways, including cytokine-cytokine receptor interaction, JAK-STAT signaling, antigen processing and presentation, Th1 and Th2 cell differentiation, and natural killer cell mediated cytotoxicity (Figure S2A). Notably, several pathways associated with antiviral responses, such as Coronavirus disease-COVID-19, Epstein–Barr virus infection, and Herpes simplex virus 1 infection, were also enriched, reflecting the activation of broad-spectrum host defense programs. Conversely, downregulated genes were significantly enriched in multiple metabolic pathways, including fatty acid metabolism, AMPK signaling pathway, bile secretion, retinol metabolism, and biosynthesis of cofactors (Figure S2B).

Consistently, Gene Set Enrichment Analysis (GSEA) showed significant positive enrichment of the inflammatory response gene set, whereas the fatty acid metabolism pathway was significantly negatively enriched in the SL1344 group (FDR < 0.01) (Figure 2C). Collectively, these results indicate that SL1344 infection induces coordinated activation of immune responses while suppressing metabolic processes in the colon, thereby establishing a transcriptional basis for subsequent evaluation of Y40-mediated protection.

### 3.3. Y40 Pretreatment Reprograms Host Transcriptional Responses Toward Metabolic Homeostasis

To determine how Y40 modulates host responses during *Salmonella* infection, gene expression profiles were compared between the SL1344 and Y40+SL1344 groups. Venn analysis identified 17,996 commonly expressed genes, with 600 and 2108 genes uniquely expressed in the Y40+SL1344 and SL1344 groups, respectively. A total of 1113 DEGs, including 207 upregulated genes and 906 downregulated genes in the Y40+SL1344 group relative to the SL1344 group, were identified (Figure 2D, Table S2), indicating that Y40 markedly reshapes infection-induced transcriptional responses.

GO enrichment analysis showed that the immune-related processes, including inflammatory response, immune response, cytokine-mediated signaling, and innate immune response, which were activated by *Salmonella* infection as described above, were suppressed in the Y40+SL1344 group compared with the SL1344 group (Figure 2E). In contrast, metabolic and biosynthetic processes were enriched among upregulated genes, including fatty acid metabolism, bile acid biosynthesis, glutathione derivative biosynthesis, and cholesterol transport, suggesting a coordinated shift from inflammatory activation toward metabolic recovery. On the other hand, metabolic pathways, such as drug metabolism-cytochrome P450, steroid hormone biosynthesis, retinol metabolism, and glutathione metabolism, were significantly upregulated (Figure S3A). Conversely, downregulated genes were significantly enriched in immune-related pathways, including cytokine-cytokine receptor interaction, Th17 cell differentiation, and Inflammatory bowel disease, reflecting Y40-mediated suppression of infection-induced inflammatory programs (Figure S3B). GSEA further confirmed these trends, showing positive enrichment of fatty acid metabolism and negative enrichment of inflammatory response gene sets in the Y40+SL1344 group (FDR < 0.01) (Figure 2F). Together, these results demonstrate that Y40 pretreatment alleviates infection-induced inflammation while restoring metabolic homeostasis at the transcriptional level.

**Figure 3:**
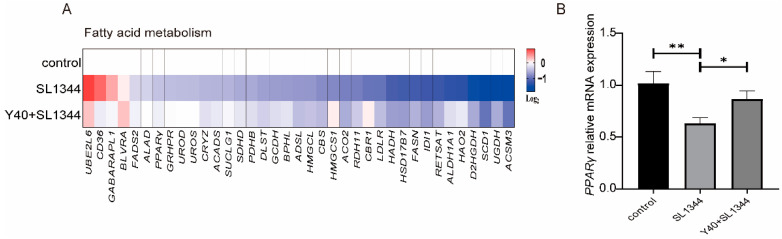
Y40 pretreatment reprograms fatty acid metabolism-related transcriptional responses during *S.* Typhimurium SL1344 infection. (**A**) Heatmap showing expression levels of key fatty acid metabolism genes across groups. (**B**) Relative *PPARγ* mRNA expression in colonic tissues determined by qRT-PCR (normalized to *β-actin*). *n* = 3 per group. ** *p* < 0.01; * *p* < 0.05 (Kruskal–Wallis test with Dunn’s post hoc test). Error bars denote SEM.

Analysis of key metabolic genes revealed that SL1344 infection markedly suppressed the expression of fatty acid metabolism-related genes, including *FASN*, *HSD17B7*, *SCD1*, *ACO2*, and *HMGCS1*, while upregulating fatty acid uptake and lipid turnover-associated genes such as *CD36*, *UBE2L6*, and *GABARAPL1* (Figure 3A). In contrast, Y40 pretreatment largely reversed these changes, restoring gene expression of these fatty acid metabolism genes toward control levels.

Notably, peroxisome proliferator-activated receptor gamma (PPARγ), a master transcriptional regulator linking lipid metabolism and inflammation, was significantly downregulated following SL1344 infection (*p* < 0.01) but restored by Y40 pretreatment (*p* < 0.05) (Figure 3B), suggesting that PPARγ signaling may underlie Y40-mediated transcriptional reprogramming.

### 3.4. Y40 Restores Host Fatty Acid Profiles Disrupted by Salmonella Infection

**Figure 4:**
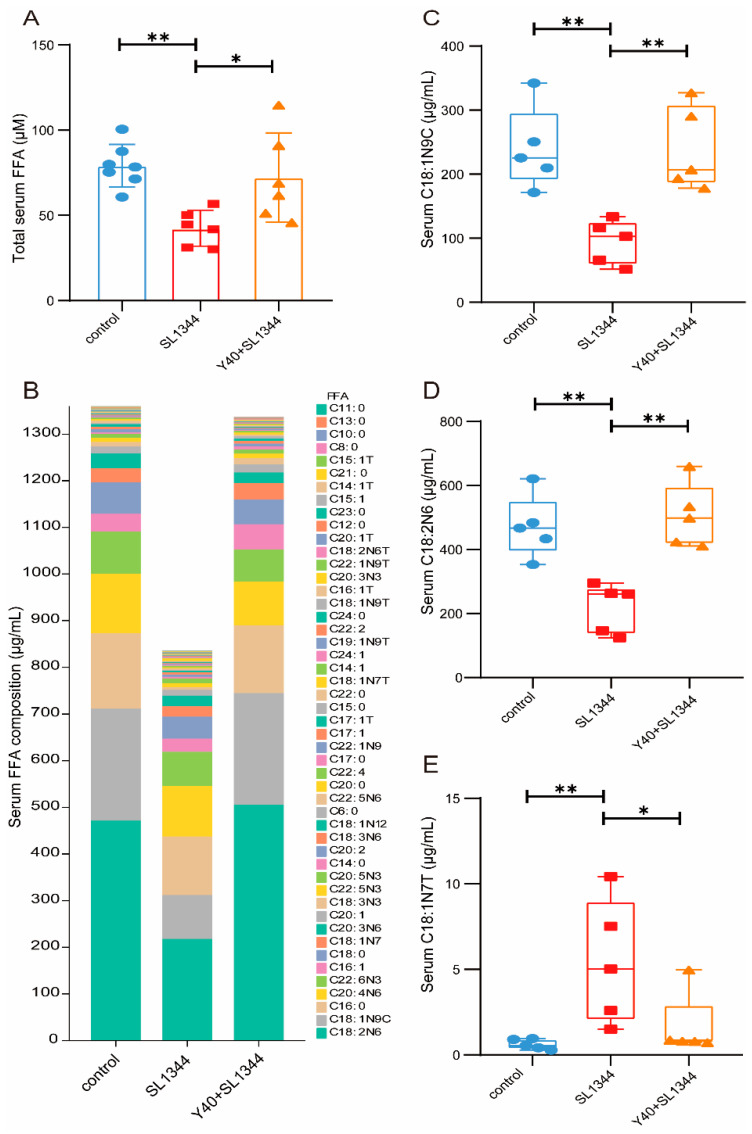
Y40 pretreatment modulates serum FFA profiles during *S.* Typhimurium SL1344 infection. (**A**) Total serum FFA concentrations. (**B**) Relative abundance of individual FFA species determined by GC-MS. (**C**–**E**) Representative fatty acids (oleic acid, linoleic acid, and trans-vaccenic acid). *n* = 5 mice per group. * *p* < 0.05; ** *p* < 0.01 (Kruskal–Wallis test with Dunn’s post hoc test).

To further assess metabolic alterations, serum free fatty acid (FFA) profiles were analyzed. SL1344 infection significantly reduced total FFA levels compared with controls (*p* < 0.01), whereas Y40 pretreatment significantly restored FFA concentrations to near-control levels (*p* < 0.05) (Figure 4A). Targeted metabolomic analysis (GC-MS) of 51 FFA species revealed substantial alterations of fatty acid composition following SL1344 infection (Figure 4B). Levels of key saturated (palmitic acid, C16:0; stearic acid, C18:0), monounsaturated (oleic acid, C18:1N9C; elaidic acid, C18:1N9T; and cis-10-heptadecenoic acid, C17:1), and polyunsaturated fatty acids (arachidonic acid, C20:4N6; linoleic acid, C18:2N6; trans-linoleic acid, C18:2N6T; and α-linolenic acid, C18:3N3) were significantly decreased in the SL1344 group, while Y40 pretreatment effectively reversed these changes (Figure 4C,D and Tables S3 and S4). In contrast, trans-vaccenic acid (C18:1N7T) was significantly elevated in the SL1344 group, and this increase was also reversed by Y40 pretreatment (*p* < 0.05) (Figure 4E).

### 3.5. Y40-Mediated Protection Depends on PPARγ-Mediated Regulation of Fatty Acid Metabolism

**Figure 5:**
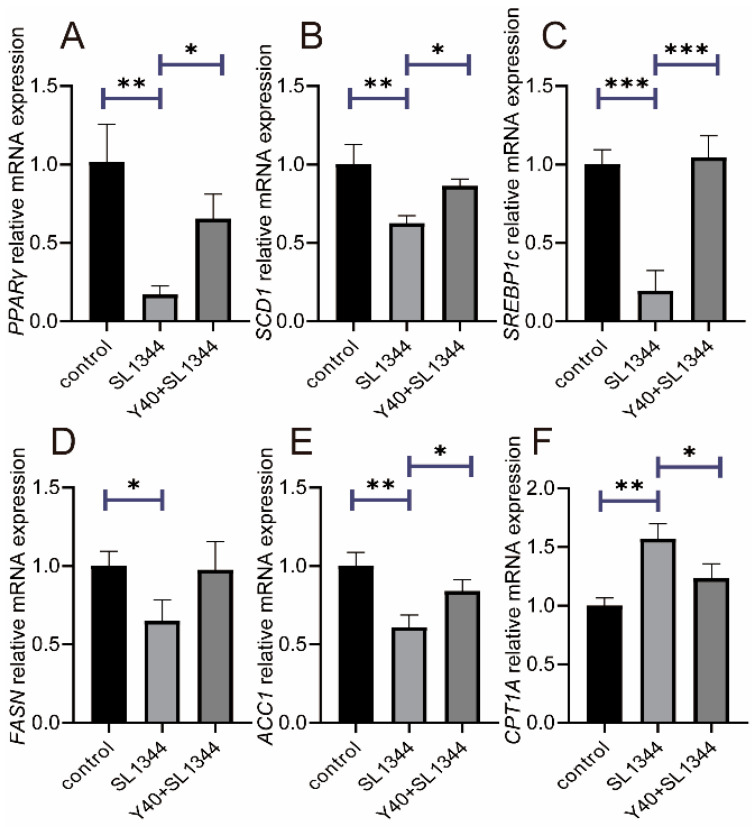
Y40 pretreatment activates *PPARγ* expression and restores lipid metabolic gene expression in MC38 cells. (**A**–**F**) qRT-PCR analysis of *PPARγ*, *SCD1*, *SREBP1c*, *FASN*, *ACC1*, and *CPT1A* (normalized to *β-actin*). Data are from three independent experiments performed in triplicate. * *p* < 0.05; ** *p* < 0.01; *** *p* < 0.001 (Kruskal–Wallis test with Dunn’s post hoc test). Error bars denote SD.

To validate the role of PPARγ, an in vitro infection model using MC38 cells was established. Consistent with in vivo results, SL1344 infection significantly downregulated *PPARγ* expression (*p* < 0.001), along with several key genes involved in fatty acid synthesis, including *SCD1*, *SREBP1c, FASN*, and *ACC1* (*p* < 0.05 for all) (Figure 5A–E). Y40 pretreatment significantly restored the expression of these genes (*p* < 0.05). In contrast, CPT1A, which catalyzes the rate-limiting step of mitochondrial fatty acid β-oxidation, was significantly upregulated in response to SL1344 infection (*p* < 0.01), and Y40 intervention normalized *CPT1A* expression levels (*p* < 0.05) (Figure 5F).

**Figure 6:**
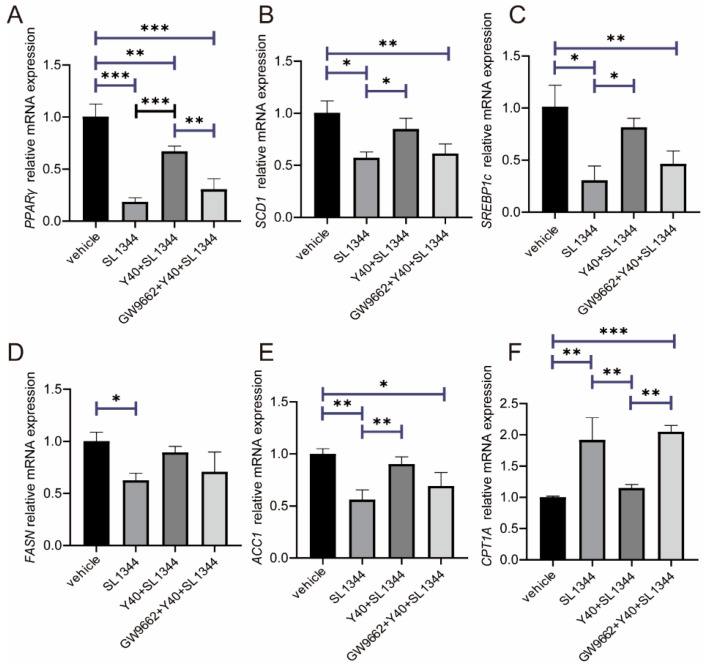
Inhibition of PPARγ abolishes Y40-mediated restoration of fatty acid metabolism genes. MC38 cells treated with vehicle, SL1344, Y40+SL1344, or GW9662+Y40+SL1344. (**A**–**F**) qRT-PCR analysis of *PPARγ*, *SCD1*, *SREBP1c*, *FASN*, *ACC1*, and *CPT1A* (normalized to *β-actin*). * *p* < 0.05; ** *p* < 0.01; *** *p* < 0.001.

**Figure 7:**
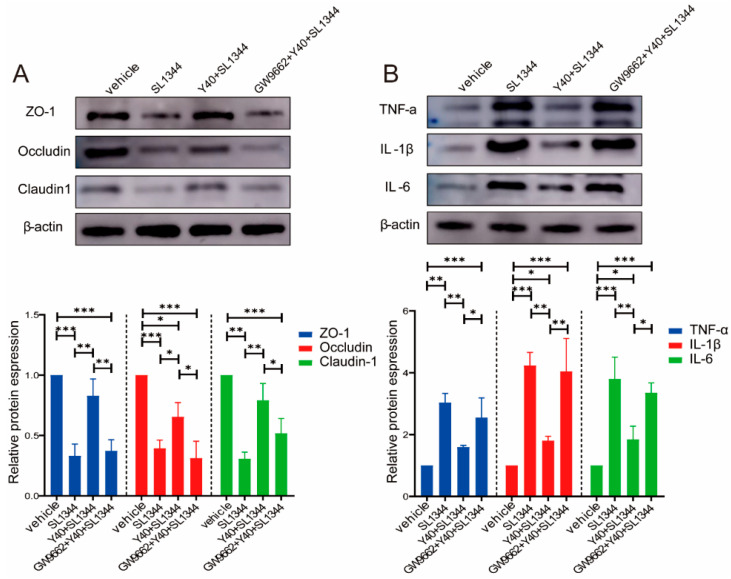
PPARγ activation is required for Y40-mediated barrier protection and anti-inflammatory effects. (**A**) Representative Western blot (**top**) and quantification (**bottom**) of tight junction proteins (ZO-1, Occludin, Claudin-1). (**B**) Representative Western blot (**top**) and quantification (**bottom**) of pro-inflammatory cytokines (TNF-α, IL-1β, IL-6). β-actin served as loading control. Data represent mean ± SD from three independent experiments. * *p* < 0.05; ** *p* < 0.01; *** *p* < 0.001.

To determine causality, the PPARγ antagonist GW9662 was applied. As shown in Figure 6A–F, pharmacological inhibition of PPARγ significantly abolished Y40-induced restoration of *PPARγ* (*p* < 0.001), *SCD1* (*p* < 0.05), and *CPT1A* (*p* < 0.001), with expression levels comparable to those in the SL1344 group (*p* > 0.05). GW9662 also tended to attenuate the restorative effects of Y40 on *SREBP1c*, *FASN*, and *ACC1*. Furthermore, Y40-mediated restoration of epithelial tight junction proteins (ZO-1, Occludin, and Claudin-1) was impaired by GW9662 (Figure 7A). Similarly, the suppressive effects of Y40 on pro-inflammatory cytokines (TNF-α, IL-1β, and IL-6) were abolished upon PPARγ inhibition (Figure 7B), with cytokine levels returning to those observed in the SL1344 group. Collectively, these results demonstrate that Y40 exerts its protective effects by restoring fatty acid metabolism and suppressing inflammation through a PPARγ-dependent mechanism.

## 4. Discussion

The intestinal epithelium constitutes a critical physical and immunological barrier against enteric pathogens [45]. *Salmonella* Typhimurium, a major cause of bacterial gastroenteritis, disrupts this barrier by inducing host inflammatory responses that simultaneously facilitate pathogen expansion and reshape the intestinal microenvironment [46,47]. In the present study, SL1344 infection elicited a canonical host response characterized by robust inflammatory activation and metabolic suppression, confirming the successful establishment of the infection model. Building on this, we demonstrate that *L. plantarum* Y40 confers protection against *Salmonella* infection by restoring host fatty acid metabolism in a PPARγ-dependent manner, thereby preserving intestinal barrier integrity and limiting excessive inflammation.

The interplay between host metabolism and pathogen infection is increasingly recognized as a key determinant of disease progression [48]. Pathogens actively reprogram host metabolic pathways to create conditions favorable for their survival [49]. *Salmonella*, for instance, exploits inflammation-derived metabolites such as nitrate to support its respiration, while disrupting commensal-mediated colonization resistance [50]. Among these processes, lipid metabolism is particularly critical, as it underpins epithelial integrity and immune homeostasis [51,52]. In this study, transcriptomic analysis revealed that SL1344 infection induced a marked shift toward an immune-dominant transcriptional state, with activation of inflammatory pathways (e.g., cytokine-cytokine receptor interaction and JAK-STAT signaling) and concurrent suppression of metabolic pathways, including fatty acid metabolism and bile acid synthesis. This reprogramming was further validated at the functional level by reduced circulating free fatty acids (FFAs) and altered lipid composition. Notably, key fatty acids such as oleic acid (C18:1N9C) and linoleic acid (C18:2N6) were significantly depleted, indicating disruption of lipid-mediated regulatory networks. Together, these findings support the concept that *Salmonella* infection drives a coordinated immunometabolic shift that compromises host homeostasis [53].

Probiotic-mediated protection against enteric infection has traditionally been attributed to direct antimicrobial activity, competitive exclusion, and immunomodulation [54,55,56]. However, emerging evidence suggests that modulation of host metabolism represents an additional, and potentially central, mechanism [57]. In this context, our study identifies host lipid metabolism rather than solely immune signaling as a primary target of probiotic intervention. Specifically, Y40 pretreatment reversed infection-induced transcriptional reprogramming by suppressing excessive immune activation while restoring metabolic pathways, particularly those related to fatty acid metabolism.

PPARγ emerges from our data as a key integrator of these effects. As a master regulator of lipid metabolism and inflammation, PPARγ governs the balance between fatty acid synthesis, uptake, and oxidation, while simultaneously exerting anti-inflammatory functions [58,59]. Consistent with previous studies, we observed that SL1344 infection significantly suppressed PPARγ expression and its downstream targets, including FASN, ACC1, SCD1, and SREBP1c, reflecting impaired lipid anabolic capacity [60]. Y40 pretreatment restored PPARγ expression and reactivated its transcriptional network, suggesting that probiotic intervention re-engages host metabolic control systems disrupted during infection [61]. These findings position PPARγ as a central node linking metabolic recovery to epithelial integrity and immune regulation during infection.

The restoration of specific fatty acid species by Y40 provides additional mechanistic insight. Among these, oleic acid (C18:1N9C) is particularly noteworthy because it functions not only as a major membrane lipid component but also as an endogenous activator of PPARγ signaling [62,63]. Previous studies have shown that oleic acid can enhance epithelial barrier integrity and suppress excessive inflammatory signaling by promoting PPARγ-dependent transcriptional programs [64,65,66]. Reduced oleic acid levels during *Salmonella* infection may therefore contribute to impaired epithelial homeostasis and exaggerated inflammatory responses, whereas Y40-mediated restoration of oleic acid could help re-establish metabolic and barrier balance. Similarly, linoleic acid (C18:2N6) and α-linolenic acid (C18:3N3), which were markedly decreased following SL1344 infection and restored by Y40 pretreatment, are essential polyunsaturated fatty acids involved in the generation of bioactive lipid mediators associated with inflammation resolution and mucosal repair [67,68,69]. Depletion of these fatty acids has been linked to disruption of tight-junction integrity and increased intestinal permeability under inflammatory conditions [70]. Restoration of these lipid species by Y40 may therefore contribute to maintenance of epithelial barrier function and attenuation of inflammatory injury during infection. In addition, Y40 reversed the *Salmonella*-induced increase in trans-vaccenic acid (C18:1N7T), suggesting broader remodeling of host lipid metabolic balance. Although the precise mechanism by which Y40 regulates host fatty acid composition remains unclear, several possibilities may be considered. Certain *Lactiplantibacillus* strains are capable of producing bioactive fatty acid derivatives, including conjugated linoleic acids and other lipid metabolites that can directly or indirectly activate PPARγ signaling [71,72]. Alternatively, Y40 may modulate host lipid metabolism through upstream metabolic regulators such as AMPK or SIRT1 pathways, thereby restoring fatty acid synthesis and utilization during infection.

Compared with previously reported probiotic strains, the immunoprotective effects of *Lactobacillus* and *Bifidobacterium* species against enteric infection or intestinal inflammation have been widely attributed to modulation of cytokine signaling, Toll-like receptor pathways, or epithelial barrier reinforcement. For example, *L. casei* and *L. acidophilus* have been shown to attenuate inflammatory responses through TGF-β- and NF-κB-related pathways, while *Bifidobacterium* species, such as *B. longum*, primarily exert protective effects via VDR- or STAT3-mediated immune regulation [73,74,75]. In addition, multi-strain probiotics such as VSL#3 have been reported to enhance local fatty acid-derived metabolites, including conjugated linoleic acid, thereby indirectly influencing PPARγ activity in colitis models [61,76]. In contrast, this study extends these observations by demonstrating that the wild-derived *L. plantarum* Y40 directly links probiotic intervention to systemic and colonic fatty acid restoration, which is tightly coupled with PPARγ activation during *Salmonella* infection. Importantly, the combined evidence from transcriptomic profiling, serum fatty acid analysis, and pharmacological inhibition (GW9662) supports a causal role of the fatty acid-PPARγ axis in mediating Y40-induced protection. This places Y40 within a category of probiotics that regulate host immunometabolism through direct lipid-nuclear receptor signaling rather than solely immune modulation.

Notably, the present study did not include a Y40-only treatment group for transcriptomic and metabolomic analyses. Our previous work showed that Y40 administration alone did not induce obvious physiological alterations under normal conditions but significantly enhanced host resistance to *Salmonella* infection [28]. Therefore, the current study primarily focused on elucidating the protective mechanisms of Y40 during infection. Nevertheless, inclusion of a probiotic-only group would provide additional insight into the direct effects of Y40 on host metabolism and immune homeostasis in the absence of pathogen challenge. Future studies incorporating this experimental group will further clarify the baseline regulatory functions of Y40 in healthy hosts.

## 5. Conclusions

This study demonstrates that *L. plantarum* Y40 protects against *Salmonella* Typhimurium infection by reprogramming host immunometabolism through PPARγ-dependent pathways. Specifically, Y40 pretreatment restored fatty acid metabolic pathways and serum fatty acid profiles, upregulated PPARγ and its downstream lipid-metabolic targets, preserved epithelial tight-junction proteins, and reduced excessive inflammatory cytokine responses. The GW9662 inhibition experiment further supports PPARγ as a functional mediator of the protective phenotype. These findings suggest that the fatty acid-PPARγ axis is a promising host-directed mechanism through which *L. plantarum* strains may contribute to *Salmonella* control strategies in livestock and poultry production. Of note, the BALB/c mouse model was used in this study primarily to evaluate the protective effects and host responses of *L. plantarum* Y40 during *Salmonella* infection. Therefore, although the identified immunometabolic mechanisms provide important mechanistic insights, the anti-*Salmonella* efficacy of Y40 in livestock species still requires further validation in future studies.

## Data Availability

The RNA sequencing data have been deposited in the China National GenBank database (CNGBdb) at https://db.cngb.org/search/project/CNP0009403 (accessed on 2 April 2026), under project ID CNP0009403.

## Supplementary Materials

The following supporting information can be downloaded at: https://www.mdpi.com/article/10.3390/microorganisms14061210/s1, Table S1: Differentially expressed genes between SL1344 and control groups; Table S2: DEGs between Y40+SL1344 and SL1344 groups; Table S3: FFA comparison between the SL1344 and control groups. Green denotes higher in SL1344 group and red denotes higher in control group; Table S4: FFA comparison between the SL1344 and Y40+SL1344 groups. Green denotes higher in SL1344 group and red denotes higher in Y40+SL1344 group; Table S5: Primers used in this study; Figure S1: Body weight dynamics of mice in different groups. The mice were weighed daily from 1 dpi to 6 dpi (*n* = 8 per group). Mann–Whitney U test was used to determine the difference between SL1344 and Y40+SL1344 groups; Figure S2: KEGG enrichment analyses of differentially expressed genes in colonic tissue following *S*. Typhimurium SL1344 infection. (A) KEGG enrichment analysis of upregulated infection-regulated genes. (B) KEGG enrichment analysis of downregulated infection-regulated genes. RNA-seq was performed using three colonic samples per group; Figure S3: KEGG enrichment analyses of differentially expressed genes between Y40+SL1344 and SL1344 groups. (A) Upregulated genes in Y40+SL1344. (B) Downregulated genes in Y40+SL1344. RNA-seq was performed using three colonic samples per group.
