# Supplementary material for: Lactiplantibacillus plantarum Y40 Ameliorates Salmonella Infection via PPARγ-Mediated Regulation of Fatty Acid Metabolism in Mice
Source: Microorganisms. 2026 May 27;14(6):1210. doi: 10.3390/microorganisms14061210 (PMC13302842; doi:10.3390/microorganisms14061210)
Supplement: Supplementary file 1 [file microorganisms-14-01210-s001.zip › Supplementary Material.pdf]

Supplementary Material

Fig. S1

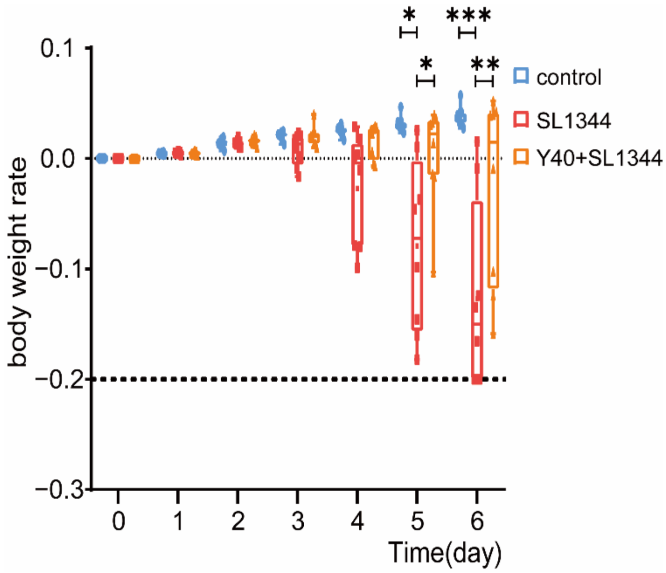

Fig. S1 Body weight dynamics of mice in different groups. The mice were weighed daily from 1 dpi to 6 dpi (n = 8 per group). Mann-Whitney U test was used to determine the difference between SL1344 and Y40+SL1344 groups.

Fig. S2

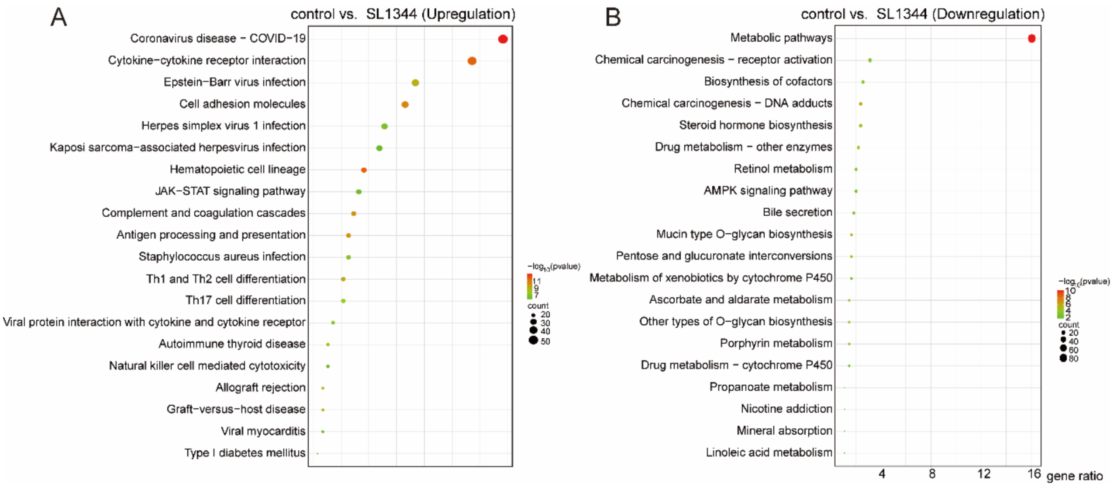

Fig. S2 KEGG enrichment analyses of differentially expressed genes in colonic tissue following *S.* Typhimurium SL1344 infection. (A) KEGG enrichment analysis of upregulated infection-regulated genes. (B) KEGG enrichment analysis of downregulated infection-regulated genes. RNA-seq was performed using three colonic samples per group.

Fig. S3

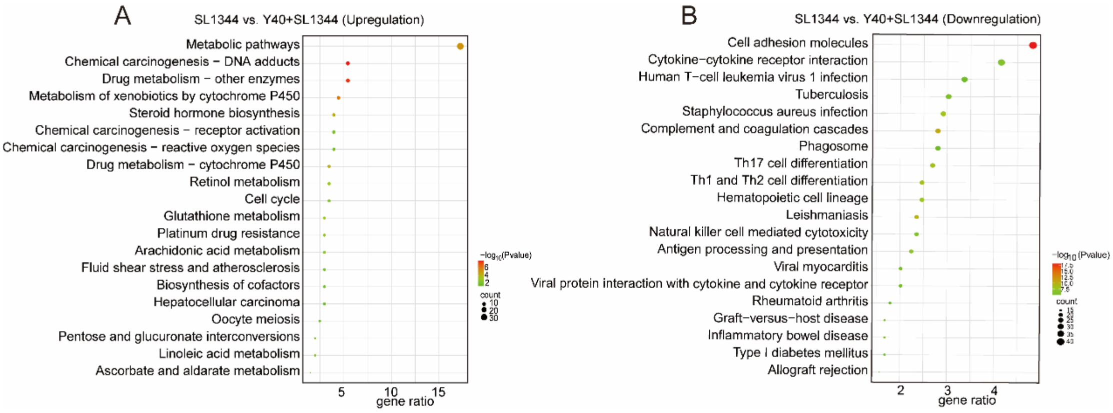

Fig. S3 KEGG enrichment analyses of differentially expressed genes between Y40+SL1344 and SL1344 groups. (A) Upregulated genes in Y40+SL1344. (B) Downregulated genes in Y40+SL1344. RNA-seq was performed using three colonic samples per group.
